# Initial Experience with MR-Guided or CT-Guided Stereotactic Body Radiotherapy for Prostate Cancer

**DOI:** 10.3390/curroncol33090504

**Published:** 2026-08-25

**Authors:** Marc Vincent N. Barcelona, Noelia Sanmamed Salgado, Enrique Gutierrez Valencia, Alejandro Berlin, Rachel Glicksman, Charles Catton, Andrew McPartlin, Jeff D. Winter, Jennifer Dang, Vickie Kong, Yangqing Deng, Peter Chung

**Affiliations:** 1 Radiation Medicine Program, Princess Margaret Cancer Centre, University of Toronto, 610 University Ave, Toronto, ON M5G 2M9, Canada; marcvincentbarcelona1@gmail.com (M.V.N.B.); jeff.winter@uhn.ca (J.D.W.); jennifer.dang@uhn.ca (J.D.);; 2 Department of Radiotherapy, Jose R. Reyes Memorial Medical Center, San Lazaro Compound, Rizal Ave. Sta Cruz, Manila 1003, Philippines; 3 Department of Radiation Oncology, Manila Doctors Hospital, 667 United Nations Avenue, Ermita, Manila 1000, Philippines; 4 Department of Radiation Oncology, San Carlos Hospital, Calle del Prof Martín Lagos, s/n, 28040 Madrid, Spain; 5 Department of Radiation Oncology, Dr. H. Bliss Murphy Cancer Centre, Memorial University of Newfoundland, 300 Prince Philip Drive, St. John’s, NL A1B 3V6, Canada; 6 Department of Biostatistics, University Health Network, 610 University Ave, Toronto, ON M5G 2M9, Canada

**Keywords:** prostate cancer, stereotactic body radiation therapy, stereotactic ablative body radiotherapy, ultrahypofractionation, MR-guided radiation therapy, adaptive radiotherapy

## Abstract

We report early toxicity outcomes after institutional implementation of prostate SBRT using 42.7 Gy in seven fractions delivered with either MR-guided adaptive SBRT (MRgSBRT) or CT-guided non-adaptive SBRT (CTgSBRT). Between July 2019 and May 2023, 216 patients received this regimen. Genitourinary (GU) and gastrointestinal (GI) toxicities were retrospectively assessed using the Common Terminology Criteria for Adverse Events (CTCAE) version 5.0. Most patients had intermediate-risk disease (86%), and 14% had high-risk disease. Median prostate volume was 40 mL, and median baseline International Prostate Symptom Score (IPSS) was 8 among patients with available data. MRgSBRT and CTgSBRT were used in 100 (46%) and 116 (54%) patients, respectively. At a median follow-up of 19 months, 12- and 24-month grade ≥ 2 GU toxicity rates were 20.0% and 18.2%, while grade ≥ 2 GI toxicity rates were 3.5% and 3.6%, respectively. No statistically significant platform-related differences were detected; however, these comparisons were exploratory and potentially confounded by nonrandomized platform selection, treatment era, rectal-spacer use, adaptive workflow and differing PTV margins. On univariable analysis, baseline GU symptoms were associated with higher 12-month grade ≥ 2 GU toxicity (OR, 3.68; 95% CI, 1.52–10.37; *p* = 0.007), while prostate-volume category was not significantly associated with this endpoint.

## Simple Summary

Prostate stereotactic body radiation therapy (SBRT) using an ultrahypofractionated regimen provides a shorter course of radiotherapy for localized prostate cancer. In this institutional cohort, treatment using either MR-guided adaptive SBRT or CT-guided non-adaptive SBRT was associated with acceptable short- and intermediate-term toxicity. Baseline urinary symptoms were associated with higher 12-month grade ≥ 2 genitourinary toxicity, while prostate-volume category was not significantly associated with this endpoint. Because treatment platform was not randomized and differed in treatment era, margin selection, rectal-spacer use and adaptive workflow, this study was not designed to establish equivalence between treatment platforms.

## 1. Introduction

A recent projection estimates that prostate cancer (PC) rates will double in the next 20 years and, with increasing incidence, there is an increasing demand for health care systems to provide necessary treatment, perhaps without a substantial increase in resources [1]. External beam radiotherapy is a safe and effective treatment modality for PC.

Recently, ultrahypofractionated radiotherapy (UHFRT) has been established to be a safe and effective treatment regimen and has comparable outcomes with longer fractionations while being more efficient and convenient for both patients and health systems. The HYPO-RT-PC trial utilized 42.7 Gy in seven fractions given every other day and showed non-inferior biochemical control (BC) and long-term toxicities compared to conventionally fractionated RT [2]. The PACE-B trial demonstrated that five-fraction SBRT was noninferior to conventional or moderately hypofractionated radiotherapy for biochemical or clinical failure at 5 years [3]. A separate two-year toxicity analysis reported similar RTOG grade ≥ 2 GU and GI toxicity between treatment groups [4]. SBRT using an ultrahypofractionated dose has been adopted as a standard treatment option for low- and intermediate-risk prostate cancer, while its use in selected high-risk patients remains more individualized [5]. More recently, PACE-C extended randomized safety evidence to patients with intermediate- and selected high-risk disease receiving short-course androgen deprivation therapy; early RTOG grade ≥ 2 GU and GI toxicity was similar between five-fraction SBRT and 20-fraction moderately hypofractionated radiotherapy, although mature disease-control results remain pending [6].

Magnetic Resonance-guided SBRT in PC has been shown to reduce short- and intermediate-term toxicities compared to CT-guided SBRT in the MIRAGE trial [7]. Daily adaptive radiotherapy may further reduce toxicity by accounting for interfraction anatomical variation, although the magnitude of benefit in prostate SBRT remains under investigation [8]. The implementation of MR-guided adaptive prostate SBRT involves several changes beyond the imaging platform itself, including daily online recontouring and reoptimization, potentially smaller planning margins and different approaches to intrafraction motion assessment. Consequently, real-world comparisons with CT-guided non-adaptive treatment are vulnerable to confounding by treatment era, margin selection, rectal-spacer use and patient selection. Additional institutional data are therefore useful for describing the feasibility and toxicity of specific fractionation regimens across evolving treatment workflows, while recognizing that nonrandomized comparisons cannot establish platform equivalence or superiority.

We adopted ultrahypofractionated radiotherapy and subsequently implemented online adaptive treatment. In this study, we describe our institutional implementation and short- to intermediate-term toxicity outcomes among patients treated with 42.7 Gy in seven fractions using either a 1.5 T magnetic resonance linear accelerator with daily adaptation or a CT-guided non-adaptive linear accelerator. We also explore clinical and treatment-related factors associated with GU and GI toxicity. The study was not designed to establish comparative equivalence or superiority between the treatment platforms.

## 2. Materials and Methods

### 2.1. Patients and Treatment

PC patients treated with 42.7 Gy in seven fractions every other day using either CT or MR guidance from 1 July 2019 to 31 May 2023 were included in this retrospective cohort. Selection for treatment using the seven-fraction SBRT regimen was individualized by the treating radiation oncologist. Clinical considerations included baseline urinary function, prostate volume, prostate and pelvic anatomy, disease-risk category, treatment feasibility, relevant comorbidities and patient preference. Selected patients with high-risk disease were treated using the seven-fraction regimen following individualized clinical assessment, consistent with randomized evidence supporting 42.7 Gy in seven fractions in intermediate- and high-risk disease [2]. The seminal vesicles were included as clinically indicated, and elective pelvic nodal irradiation was not delivered. The institutional seven-fraction SBRT protocol did not specify absolute prostate-volume or baseline-IPSS exclusion thresholds; treatment selection was individualized by the treating radiation oncologist. The use of CTgSBRT or MRgSBRT, fiducial markers, rectal spacers, and androgen deprivation therapy (ADT) were at the treating physician’s discretion and consistent with institutional policy. Rectal-spacer placement was not mandatory and was determined individually. During the study period, publicly funded indications included inflammatory bowel disease, ongoing anticoagulant use and bilateral hip prostheses. In patients without these indications, spacer placement could be considered after discussion of the potential risks and benefits, patient preference and any associated out-of-pocket costs. ADT use was recorded as a binary variable; however, ADT duration and sequencing were not reliably available and were therefore not analyzed.

Patients treated with CTgSBRT underwent CT and MRI simulation. The simulation T2-weighted MRI was fused with the planning CT, and the apparent diffusion coefficient map generated from diffusion-weighted imaging was also fused when requested by the treating radiation oncologist. The whole prostate gland was defined as the clinical target volume (CTV) with inclusion of the proximal half of the seminal vesicles for unfavorable intermediate-risk PC patients and the entire seminal vesicles for high-risk PC patients.

The CTV-to-PTV margin was 6 mm isotropically in patients treated without fiducial markers and 5 mm isotropically when fiducial markers were used. Patients were generally instructed to drink approximately 300 mL of water 45–60 min before simulation and each CTgSBRT treatment. Preparation was modified when clinically required to obtain an adequately filled bladder and empty rectum. An empty-rectum protocol was used without routine prophylactic laxative administration.

Cone-beam CT was obtained before every CTgSBRT fraction for soft-tissue prostate localization or fiducial-based matching, followed by online positional correction. Fiducial markers were not routinely required because soft-tissue prostate matching on CBCT was considered adequate for daily target localization. A second verification CBCT was not routinely obtained. If manual adjustments greater than 10 mm were required, the planner, radiation oncologist, and physicist were notified; when the overall displacement was ≥20 mm, a second CBCT was acquired for verification. No endorectal balloons were used, and no imaging was performed during beam delivery.

Patients treated with MRgSBRT received treatment on a 1.5 T MR-Linac using an online adapt-to-shape workflow. A daily localization MRI was acquired, after which the target and adjacent organs at risk were reviewed and recontoured as required, and the treatment plan was fully reoptimized on the anatomy of the day. During the subsequent plan quality-control interval, cine MRI was used to assess target position. A verification MRI was then acquired immediately before beam delivery to confirm that the CTV remained within the PTV. If the CTV moved outside the PTV, treatment delivery was withheld; if displacement persisted, the treatment session was aborted and restarted. A beam-on MR acquisition was obtained for dose-accumulation purposes, but cine MRI was not used for real-time intrafraction monitoring during beam delivery. A 5 mm isotropic CTV-to-PTV margin was used during the initial implementation period. Following institutional dose-accumulation quality-improvement work evaluating PTV margin design, the margin was reduced in July 2021 to 4 mm in the anteroposterior and superior–inferior directions and 3 mm laterally [9]. Platform-specific simulation, planning, image-guidance, and treatment-delivery characteristics are summarized in Supplementary Table S1.

### 2.2. Toxicity and Outcomes

Toxicity information was retrospectively abstracted from clinical documentation in the Epic electronic medical record. One investigator (M.V.N.B.) assigned Common Terminology Criteria for Adverse Events version 5.0 grades based on the documented clinical findings. The assigned grades were subsequently reviewed by a second investigator (N.S.S.). Baseline GU symptoms were recorded as present or absent based on clinical documentation, and the International Prostate Symptom Score (IPSS) was analyzed separately as both a continuous and categorical variable.

Clinical follow-up was not platform-specific; patients treated with CTgSBRT and MRgSBRT underwent routine institutional follow-up, and toxicity assessments were abstracted from available clinical encounters.

At each landmark, toxicity was assessed using two complementary measures: landmark toxicity prevalence and cumulative worst toxicity. Landmark toxicity was defined as an adverse event that was ongoing at the corresponding timepoint, based on its onset and resolution dates. Patients who had died or were lost to follow-up before a landmark were excluded from the denominator for the landmark-prevalence analysis. In contrast, cumulative worst toxicity was defined as the highest recorded grade using all available data from treatment initiation up to the corresponding landmark, loss to follow-up, or death, whichever occurred first, irrespective of whether the toxicity had subsequently resolved. All 216 patients therefore contributed a value to the cumulative-worst toxicity analysis at each landmark.

Because grade 1 toxicity could not be consistently distinguished from no toxicity in retrospective clinical documentation, grade 0–1 toxicity was not reported separately. Analyses therefore focused on grade ≥ 2 and grade ≥ 3 GU and GI toxicity.

Baseline patient and disease characteristics were compared between treatment groups using the Wilcoxon rank-sum test, chi-square test, or Fisher’s exact test, as appropriate.

Baseline IPSS was available for 97 of 216 patients. Analyses involving IPSS were conducted as exploratory available-case analyses without imputation.

Univariable logistic regression analyses were performed to identify factors associated with grade ≥ 2 GU and GI toxicity at 12 months among patients evaluable at that landmark. Variables assessed included age, T stage, prostate-volume category, treatment platform, ADT use, baseline GU symptoms and baseline IPSS; rectal-spacer use was additionally assessed for GI toxicity. Variables with *p* < 0.05 on univariable analysis were planned for inclusion in a multivariable model. Because only one variable met this criterion, multivariable modeling was not performed for grade ≥ 2 toxicity. Because of the low number of grade ≥ 3 events, valid regression models could not be fitted for these endpoints; grade ≥ 3 outcomes were therefore summarized descriptively.

Statistical analyses were performed using R version 4.4.1. All statistical tests were two-tailed, with *p* < 0.05 considered statistically significant.

## 3. Results

The cohort included 216 patients, of whom 116 received CTgSBRT and 100 received MRgSBRT. Most patients had intermediate-risk disease. Baseline characteristics were generally similar between treatment platforms, although rectal spacers were more frequently used in the MRgSBRT cohort. Baseline IPSS was available for 97 patients. Additional patient and treatment characteristics are presented in Table 1.

**Figure 1:**
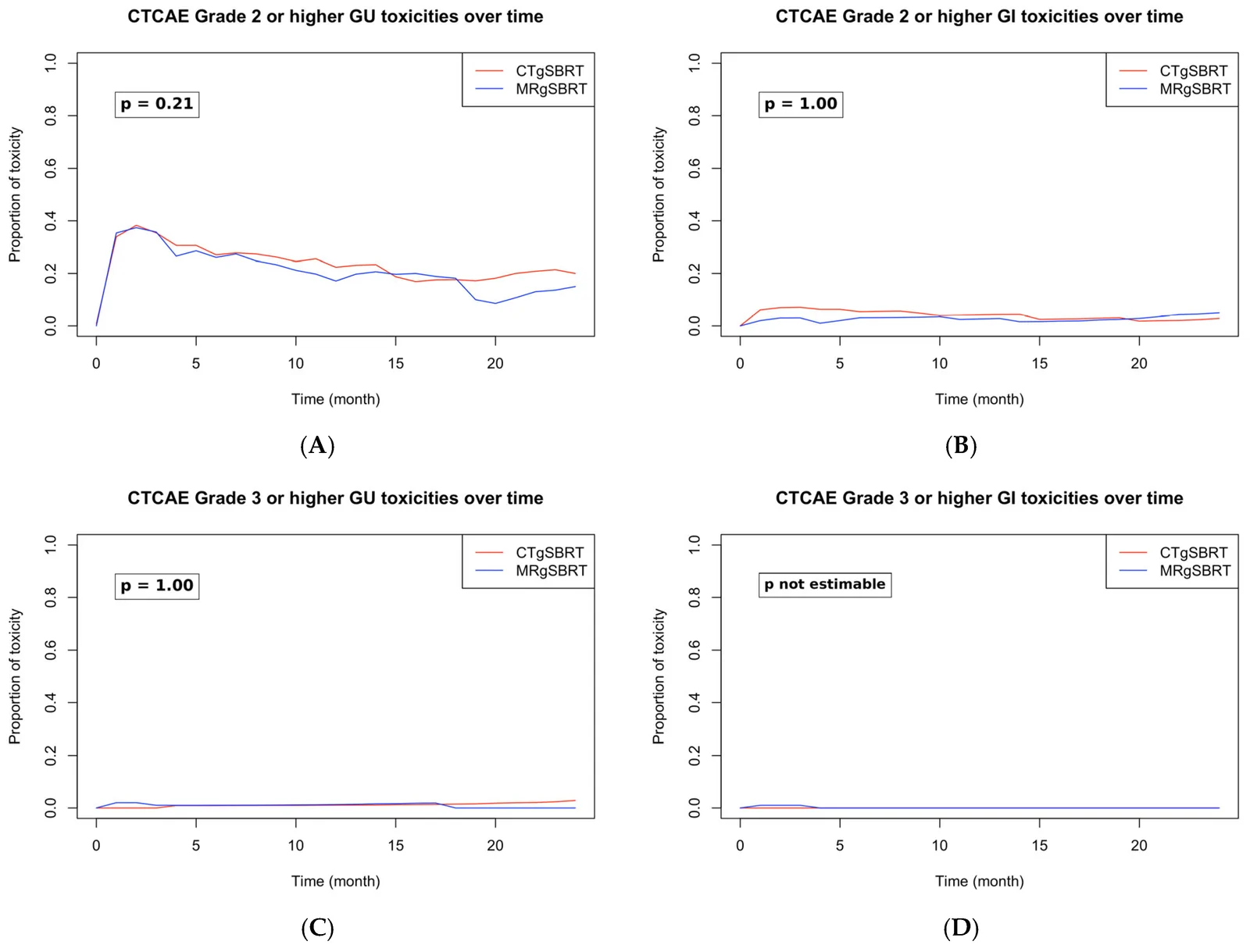
Proportion of evaluable patients with GU and GI toxicity present at each monthly landmark, stratified by treatment platform. (**A**) Grade ≥ 2 GU toxicity; (**B**) Grade ≥ 2 GI toxicity; (**C**) Grade ≥ 3 GU toxicity; (**D**) Grade ≥ 3 GI toxicity. Abbreviations: CTCAE, Common Terminology Criteria for Adverse Events; GU, genitourinary; GI, gastrointestinal; CTgSBRT, CT-guided stereotactic body radiation therapy; and MRgSBRT, MR-guided stereotactic body radiation therapy.

Toxicity information at 3 months was available for 211 patients, including 113 treated with CTgSBRT and 98 treated with MRgSBRT. Corresponding evaluable numbers at 12 months were 170 patients (94 CTgSBRT and 76 MRgSBRT), and at 24 months were 55 patients (35 CTgSBRT and 20 MRgSBRT). At a median follow-up of 19 months, G2+ GU toxicity at 3, 12, and 24 months was 35.4%, 22.3%, and 20% for CTgSBRT, and 35.7%, 17.1%, and 15% for MRgSBRT, respectively. Corresponding G2+ GI toxicity rates were 7.1%, 4.3%, and 2.9% for CTgSBRT, and 3.1%, 2.6%, and 5% for MRgSBRT, respectively. There were no statistically significant differences between CTgSBRT and MRgSBRT in 24-month G2+ GU toxicity (*p* = 0.21), G2+ GI toxicity (*p* = 1.00), or G3+ GU toxicity (*p* = 1.00); G3+ GI toxicity was rare. Cumulative worst GU and GI toxicity through each landmark is summarized in Supplementary Table S2, while toxicity present at the monthly landmarks is provided by treatment platform in Supplementary Table S3. Longitudinal grade ≥ 2 and grade ≥3 GU and GI toxicity according to treatment platform is shown in Figure 1.

Grade ≥ 3 (G3+) toxicity was uncommon, and no grade 4–5 GU or GI toxicities were observed. G3 GU toxicity occurred in 2/216 (1%), 3/216 (1%), and 3/216 (1%) patients up to 3, 12, and 24 months, respectively. The G3 GU events consisted of urethral stricture with weak stream/dysuria (*n* = 1), dribbling/dysuria/frequency (*n* = 1), and frequency/urgency/nocturia/bladder spasms/dysuria (*n* = 1). G3 GI toxicity occurred in 0/216, 1/216 (<1%), and 1/216 (<1%) patients up to 3, 12, and 24 months, respectively; the single G3 GI event was hematochezia.

At 12 months, univariable analysis showed that baseline GU symptoms were associated with higher grade ≥ 2 GU toxicity (OR, 3.68; 95% CI, 1.52–10.37; *p* = 0.007). Prostate volume of 30–59 Ml versus <30 Ml (OR, 1.73; 95% CI, 0.68–5.05; *p* = 0.28) and ≥60 Ml versus <30 Ml (OR, 0.92; 95% CI, 0.21–3.57; *p* = 0.90) were not significantly associated with toxicity. Treatment platform was also not significantly associated with 12-month grade ≥ 2 GU toxicity (OR, 0.72; 95% CI, 0.33–1.53; *p* = 0.40). No variables were significantly associated with grade ≥ 2 GI toxicity at 12 months (Table 2). Because only one variable met the *p* < 0.05 criterion, no multivariable analysis was performed.

## 4. Discussion

This retrospective institutional study describes the implementation and toxicity outcomes of a seven-fraction prostate SBRT regimen across two treatment workflows. The regimen was associated with acceptable short- and intermediate-term toxicity, with low rates of grade ≥ 3 GU and GI events. Baseline GU symptoms were associated with higher 12-month grade ≥ 2 GU toxicity on univariable analysis, whereas prostate-volume category was not significantly associated with this endpoint. Because treatment platform was not randomized and differed with respect to calendar time, margin selection, rectal-spacer use and adaptive workflow, the study was not designed to establish equivalence or comparative superiority between CTgSBRT and MRgSBRT.

SBRT is now an established standard-of-care treatment option for clinically localized PC, supported by randomized data from HYPO-RT-PC and PACE-B showing comparable disease-control outcomes to longer fractionation schedules [2,3]. In this context, our cohort provides a contemporary institutional experience using the HYPO-RT-PC 42.7 Gy in a seven-fraction regimen delivered with modern CT-guided and MR-guided workflows. Although G2+ GU toxicity was relatively common, G3+ GU/GI toxicity was uncommon and no statistically significant toxicity differences were observed between treatment platforms.

A contemporary nonrandomized comparison of prostate SBRT delivered using a 1.5 T MR-Linac or a conventional linac similarly reported no statistically significant difference in acute grade ≥ 2 GU toxicity, with a nonsignificant trend toward lower grade ≥ 2 GI toxicity with MR-guided treatment [10].

Comparisons between CTgSBRT and MRgSBRT in this cohort should be interpreted cautiously. Treatment platform was not randomized and was potentially confounded by calendar time, patient selection, rectal-spacer use, fiducial marker use, and evolving PTV margins. Therefore, the absence of statistically significant toxicity differences should not be interpreted as evidence of equivalence between platforms or as excluding a benefit from MR guidance or adaptation. These questions are best addressed in randomized studies; our institution is currently conducting a randomized study (ASPIRE), which is evaluating adaptive versus non-adaptive prostate SBRT, with additional evaluation of MR- versus CT-adaptive workflows.

The most direct randomized benchmark for our regimen is HYPO-RT-PC, which used 42.7 Gy in seven fractions. However, several differences limit direct comparison with our cohort. HYPO-RT-PC did not permit ADT, used prostate-only CTV without seminal vesicle inclusion, and used a 7 mm CTV-to-PTV margin in all directions with image guidance based on implanted markers. In contrast, our cohort included ADT use at physician discretion, included proximal or entire seminal vesicles for selected unfavorable intermediate- and high-risk patients, and used smaller CTgSBRT and MRgSBRT margins. Toxicity grading also differed, with our study using CTCAE v5.0. Therefore, our CTCAE G2+ GU toxicity rates should be interpreted in the context of different target definitions, margins, grading systems, ADT use, and medication-prescribing thresholds. The 10-year HYPO-RT-PC update confirmed non-inferior failure-free survival and similar late toxicity with ultrahypofractionation compared with conventional fractionation [11].

Image-guided adaptive radiation therapy is an important recent development in radiation oncology. The MR-Linac integrates onboard magnetic resonance imaging with a linac, allowing superior soft-tissue visualization, improved target delineation, and near real-time assessment of OAR anatomy. These capabilities create the potential for on-table adaptive planning and may theoretically improve treatment accuracy while reducing toxicity [12,13,14,15]. This potential was supported by the randomized MIRAGE trial, which demonstrated that MR-guided SBRT reduced toxicity compared with CT-guided SBRT with fiducial markers in patients with PC [7], and by a systematic review and meta-analysis suggesting lower acute grade 2 or higher GU and GI toxicity with MR-guided adaptive SBRT compared with CT-guided SBRT [16]. Notably, the MIRAGE trial used smaller PTV margins in the MR-guided arm, although interfraction adaptation was not performed, which suggests that further toxicity reduction may be possible with adaptive workflows such as those routinely used at our institution.

The relatively high rate of CTCAE G2 GU toxicity in our cohort may partly reflect institutional prescribing patterns for urinary symptoms, as CTCAE v5.0 classifies urinary frequency, urgency, or retention requiring medical management as G2 toxicity [17]. This interpretation is supported by PACE-B, where higher CTCAE-reported GU toxicity was not fully mirrored by other toxicity or patient-reported scales and was considered potentially related to investigator interpretation and variation in prescribing thresholds [4]. In addition, differences in cohort composition and treatment characteristics, including the frequent use of ADT in our cohort compared with HYPO-RT-PC and PACE-B where ADT was not permitted, may limit direct cross-trial comparisons [4].

Additional heterogeneity may have arisen from differential use of rectal spacers, fiducial markers, and evolving PTV margins. CTgSBRT was delivered using a 6 mm isotropic margin, or a 5 mm isotropic margin when fiducial markers were used. MRgSBRT initially used a 5 mm isotropic margin. Following institutional dose-accumulation quality-improvement work evaluating PTV margin design, the MRgSBRT margin was reduced in July 2021 to 4 mm in the anteroposterior and superior–inferior directions and 3 mm laterally [9]. Only 17 MRgSBRT patients were treated with the initial 5 mm isotropic margin, so the influence of this margin change on the observed toxicity outcomes cannot be isolated. Margin selection and its evolution over time therefore represent important confounders when interpreting differences between treatment platforms [2,7,15].

In our cohort, baseline GU symptoms were associated with higher grade ≥ 2 GU toxicity at 12 months. Prostate-volume category was not significantly associated with this endpoint, despite prior reports suggesting an association between increasing prostate volume and urinary toxicity [18]. These findings emphasize the potential importance of baseline urinary function during pretreatment counseling and patient optimization. Although MRgSBRT showed numerically lower GU toxicity at some timepoints, this exploratory finding did not reach statistical significance and may reflect limited power, patient selection, margin selection, spacer use, or adaptive-planning workflow rather than a true platform effect.

From a practical perspective, this cohort reflects real-world implementation of an established seven-fraction SBRT regimen alongside newer image-guided and adaptive workflows.

These findings support continued prospective evaluation of the seven-fraction regimen and suggest that baseline urinary function may be useful when counseling and optimizing patients before treatment. Future studies should incorporate standardized toxicity ascertainment, patient-reported outcomes and mature disease-control follow-up. Randomized evaluation is also needed to distinguish the effects of treatment platform, online adaptation, margin reduction and other associated workflow differences. Emerging prospective MR-guided adaptive studies, including HERMES, have demonstrated the feasibility of increasingly abbreviated prostate SBRT regimens delivered with daily adaptive replanning, while emphasizing the need for mature late-toxicity and biochemical-control data [19].

## 5. Limitations of the Study

This study has several important limitations. First, it was retrospective and nonrandomized, introducing selection bias, information bias, and residual confounding, particularly for comparisons between CTgSBRT and MRgSBRT. Treatment platform was potentially confounded by calendar time, patient selection, rectal-spacer use, fiducial marker use, adaptive workflow, and evolving PTV margins. The difference in rectal-spacer use between treatment platforms reflects clinical selection, funding eligibility, patient preference and temporal changes in practice and represents an additional source of confounding. Second, ADT use was captured as a binary variable, but ADT duration and sequencing were not reliably available, limiting evaluation of its potential association with toxicity. Third, baseline IPSS was missing for a substantial proportion of patients, and IPSS-based analyses should therefore be considered exploratory. Toxicity grades were retrospectively reconstructed from clinical documentation rather than prospectively entered at standardized CTCAE assessment visits, creating the potential for misclassification and incomplete ascertainment. Fourth, CTCAE G2+ GU toxicity may be influenced by institutional prescribing thresholds for urinary medications, because pharmacologic intervention distinguishes G2 from G1 toxicity for some urinary adverse events. The number of patients evaluable for toxicity decreased at later landmarks, particularly at 24 months, resulting in wider uncertainty around late estimates. Finally, the median follow-up of 19 months limited robust assessment of late toxicity.

## 6. Conclusions

This retrospective institutional experience describes the implementation of prostate SBRT using 42.7 Gy in seven fractions with contemporary CT-guided non-adaptive and MR-guided adaptive workflows. Treatment was associated with acceptable short- and intermediate-term toxicity, although nonrandomized platform selection and workflow differences preclude comparative conclusions regarding treatment platform. Baseline GU symptoms were associated with higher 12-month grade ≥ 2 GU toxicity on univariable analysis. Longer follow-up and prospective randomized data are required to better define late toxicity and the independent effects of online adaptation, image-guidance platform and margin reduction.

## Figures and Tables

**Table 1:** Baseline demographic characteristics of the patient cohort.

	Full Sample (*n* = 216)	CTgSBRT (*n* = 116)	MRgSBRT (*n* = 100)	*p*-Value
**Age**				0.11
Mean (SD)	73.5 (6.8)	74.2 (6.6)	72.7 (6.9)	
**T stage**				0.79
T1	105 (49%)	56 (48%)	49 (50%)	
T2	100 (47%)	54 (47%)	46 (47%)	
T3	9 (4%)	6 (5%)	3 (3%)	
Missing	2	0	2	
**NCCN Risk Stratification**				0.66
Favorable Intermediate Risk	44 (20%)	23 (20%)	21 (21%)	
Unfavorable Intermediate Risk	141 (65%)	74 (64%)	67 (67%)	
High Risk	31 (14%)	19 (16%)	12 (12%)	
**ISUP group**				0.28
Grade Group 2	157 (76%)	86 (78%)	71 (73%)	
Grade Group 3	33 (16%)	13 (12%)	20 (21%)	
Grade Group 4	7 (3%)	5 (5%)	2 (2%)	
Grade Group 5	10 (5%)	6 (5%)	4 (4%)	
Missing	9	6	3	
**PSA**				0.90
Median (IQR)	9.4 (6.9, 12.9)	9.3 (6.8, 12.9)	9.6 (7.0, 12.9)	
**Prostate volume category**				0.69
<30 mL	59 (28%)	34 (30%)	25 (26%)	
30–59 mL	112 (54%)	60 (54%)	52 (54%)	
≥60 mL	37 (18%)	18 (16%)	19 (20%)	
Missing	8	4	4	
**ADT**				0.41
No	122 (56%)	69 (59%)	53 (53%)	
Yes	94 (44%)	47 (41%)	47 (47%)	
**Baseline GU symptoms**				0.90
No	82 (38%)	45 (39%)	37 (37%)	
Yes	134 (62%)	71 (61%)	63 (63%)	
**Baseline IPSS**				
Median (IQR)	8 (4, 11)	7 (4, 12)	8.5 (4.0, 10.2)	
Missing	119	63	56	
**Baseline IPSS category**				
Mild (0–7)	47 (48%)	26 (49%)	21 (48%)	
Moderate (8–19)	45 (46%)	25 (47%)	20 (45%)	
Severe (20–35)	5 (5%)	2 (4%)	3 (7%)	
Missing	119	63	56	
**Rectal Spacer**				0.004
No	189 (88%)	109 (94%)	80 (80%)	
Yes	27 (12%)	7 (6%)	20 (20%)	

**Note:** Baseline IPSS was available for 97/216 patients; IPSS comparisons were exploratory available-case analyses. No imputation was performed. Abbreviations: SD: Standard deviation; NCCN: National Comprehensive Cancer Network; PSA: Prostate Specific Antigen; ADT: Androgen Deprivation Therapy; GU: Genitourinary; IPSS: International Prostate Symptom Score; CTgSBRT: CT-guided Stereotactic Body Radiation Therapy; and MRgSBRT: MR-guided Stereotactic Body Radiation Therapy.

**Table 2:** (**A**) Univariable analysis for grade ≥ 2 genitourinary toxicity at 12 months. (**B**) Univariable analysis for grade ≥ 2 gastrointestinal toxicity at 12 months.

(**A**)		
**Variable**	**OR (95% CI)**	***p*** **-value**
Age	1.00 (0.95–1.06)	0.91
T1	Reference	
T2 vs. T1	0.74 (0.33–1.62)	0.45
T3 vs. T1	1.20 (0.17–5.75)	0.84
Prostate volume < 30 mL	Reference	
30–59 mL	1.73 (0.68–5.05)	0.28
≥60 mL	0.92 (0.21–3.57)	0.90
MRgSBRT vs. CTgSBRT	0.72 (0.33–1.53)	0.40
ADT Yes vs. No	1.06 (0.50–2.26)	0.88
Baseline GU symptoms: Yes vs. No	3.68 (1.52–10.37)	0.007
IPSS Moderate (vs. Mild)	2.32 (0.72–8.30)	0.17
IPSS Severe (vs. Mild)	3.87 (0.43–29.99)	0.19
(**B**)		
**Variable**	**OR (95% CI)**	***p*** **-value**
Age	0.98 (0.86–1.12)	0.71
T1	Reference	
T2 vs. T1	0.23 (0.01–1.59)	0.19
T3 vs. T1	1.6 × 10^−7^ (NA–1.4 × 10^103^)	0.99
Prostate volume < 30 mL	Reference	
30–59 mL	0.26 (0.03–1.60)	0.14
≥60 mL	3.8 × 10^−8^ (NA–1.8 × 10^89^)	0.99
MRgSBRT vs. CTgSBRT	0.82 (0.11–5.07)	0.83
ADT: Yes vs. No	1.80 (0.29–13.93)	0.53
Rectal Spacer: Yes vs. No	2.04 (0.10–14.77)	0.53

**Note:** (**A**) The analysis population comprised patients evaluable at 12 months (*N* = 170; 34 grade ≥ 2 GU events). The outcome represented GU toxicity present at the 12-month landmark. IPSS analyses were exploratory available-case analyses because baseline IPSS was available for 97/216 patients. OR, odds ratio; CI, confidence interval; GU, genitourinary; ADT, androgen deprivation therapy; IPSS, International Prostate Symptom Score; MRgSBRT, MR-guided stereotactic body radiation therapy; and CTgSBRT, CT-guided stereotactic body radiation therapy. (**B**) The analysis population comprised patients evaluable at 12 months (*N* = 170). The outcome represented GI toxicity present at the 12-month landmark. OR, odds ratio; CI, confidence interval; GI, gastrointestinal; ADT, androgen deprivation therapy; MRgSBRT, MR-guided stereotactic body radiation therapy; and CTgSBRT, CT-guided stereotactic body radiation therapy.

## Data Availability

The data presented in this study are available on reasonable request from the first author (M.V.N.B.) and the corresponding authors (N.S.S. and P.C.), due to privacy and ethical restrictions related to patient-level clinical data.

## Supplementary Materials

The following supporting information can be downloaded at: https://www.mdpi.com/article/10.3390/curroncol33090504/s1, Table S1: Platform-specific simulation, planning, image-guidance, and treatment-delivery characteristics; Table S2: Cumulative worst CTCAE version 5.0 genitourinary and gastrointestinal toxicity through 3, 12, and 24 months in the overall cohort; Table S3: Monthly landmark prevalence of grade ≥ 2 and grade ≥ 3 genitourinary and gastrointestinal toxicity by treatment platform over 24 months.
