# Supplementary material for: Initial Experience with MR-Guided or CT-Guided Stereotactic Body Radiotherapy for Prostate Cancer
Source: Curr Oncol. 2026 Aug 25;33(9):504. doi: 10.3390/curroncol33090504 (PMC13605993; doi:10.3390/curroncol33090504)
Supplement: Supplementary file 1 [file curroncol-33-00504-s001.zip › curroncol-4459975-supplementary.pdf]

Supplementary Table S1. Platform-specific simulation, planning, image-guidance, and treatment-delivery characteristics.

Characteristic	CTgSBRT	MRgSBRT
**Prescribed regimen**	42.7 Gy in 7 fractions delivered every other day	42.7 Gy in 7 fractions delivered every other day
**Clinical target volume**	Whole prostate; proximal half of the seminal vesicles for unfavorable intermediate-risk disease and entire seminal vesicles for high-risk disease	Same institutional target-definition approach
**Simulation/planning imaging**	Planning CT with fused T2-weighted MRI; apparent diffusion coefficient map from diffusion-weighted imaging fused when requested	MR-based planning workflow with registered CT-derived electron-density information
**Bladder preparation**	Approximately 300 mL water 45–60 minutes before simulation and treatment; modified when clinically required	Approximately 300 mL water about 10 minutes before simulation and treatment; modified when clinically required
**Rectal preparation**	Empty-rectum protocol without routine prophylactic laxative use	Empty-rectum protocol; preparation modified when clinically required
**Daily localization and verification**	CBCT before every fraction for soft-tissue or fiducial-based matching, followed by online positional correction. A second verification CBCT was not routinely obtained; manual adjustments >10 mm prompted notification of the planner, radiation oncologist, and physicist, and a second CBCT was obtained when overall displacement was ≥20 mm.	Daily localization MRI before online adaptation, followed by a verification MRI immediately before beam delivery to confirm that the CTV remained within the PTV
**Intrafraction motion management**	No imaging during beam delivery	Cine MRI during the plan quality-control interval was used to assess target position. If the CTV moved outside the PTV, beam delivery was withheld; persistent displacement prompted abortion and restarting of the treatment session. A beam-on MR acquisition was obtained for dose accumulation, but cine MRI was not used during beam delivery.
**Online adaptation**	None	Adapt-to-shape workflow with online review/recontouring and full inverse reoptimization before delivery
**CTV-to-PTV margin**	6 mm isotropically without fiducials; 5 mm isotropically with fiducials	Initially 5 mm isotropically (17/100 patients); from July 2021, 4 mm AP/SI and 3 mm LR (83/100 patients), based on institutional dose-accumulation quality-improvement work evaluating PTV margin design
**Fiducial markers**	Optional; not routinely required because soft-tissue CBCT matching was used for daily localization; used in 4/116 patients.	Not required for MR localization; used in 0/100 patients
**Endorectal balloon**	Not used	Not used
**Rectal spacer**	Individualized; used in 7/116 patients. Publicly funded indications included inflammatory bowel disease, ongoing anticoagulant use, and bilateral hip prostheses; otherwise considered after discussion of risks, benefits, preference, and cost	Individualized using the same policy; used in 20/100 patients
**Target-coverage planning goals**	CTV D99 >42.7 Gy; PTV D99 >40.6 Gy; PTV D1cc <44.8 Gy	CTV D99 >42.7 Gy; PTV D99 >40.6 Gy; PTV D1cc <46.1 Gy
**Rectal planning goals**	D50 <7 Gy; D20 <25 Gy; D1cc <42.7 Gy	D50 <10 Gy; D20 <24 Gy; D1cc <42.7 Gy
**Bladder planning goals**	D40 <16 Gy; D5cc <42.7 Gy	D40 <18 Gy; D5cc <42.7 Gy
**Urethral planning goal**	D50 <44 Gy	D50 <45 Gy
**Patient-level dose-constraint compliance**	Not retrospectively quantified	Not retrospectively quantified

Planning goals are protocol-specified criteria; patient-level compliance and protocol-version-specific deviations were not retrospectively quantified. Abbreviations: ADC, apparent diffusion coefficient; AP, anteroposterior; CBCT, cone-beam computed tomography; CTV, clinical target volume; Dxx, dose received by xx% of the structure; Dxcc, dose to the hottest x cm³; LR, left–right; MR, magnetic resonance; PTV, planning target volume; SI, superior–inferior.

Supplementary Table S2. Cumulative worst CTCAE version 5.0 genitourinary and gastrointestinal toxicity through 3, 12, and 24 months in the overall cohort.

	n=216<br>Values are n (%).
**Cumulative worst GU toxicity through 3 months**	
Grade 2	84 (39)
Grade ≥3	2 (1)
**Cumulative worst GU toxicity through 12 months**	
Grade 2	92 (43)
Grade ≥3	3 (1)
**Cumulative worst GU toxicity through 24 months**	
Grade 2	95 (44)
Grade ≥3	3 (1)
**Cumulative worst GI toxicity through 3 months**	
Grade 2	8 (4)
Grade ≥3	0 (0)
**Cumulative worst GI toxicity through 12 months**	
Grade 2	14 (6)
Grade ≥3	1 (<1)
**Cumulative worst GI toxicity through 24 months**	
Grade 2	14 (6)
Grade ≥3	1 (<1)

Cumulative worst toxicity was defined as the highest recorded grade using all available data from treatment initiation up to each landmark, loss to follow-up, or death, whichever occurred first. Toxicities occurring and resolving before a landmark were included. All 216 patients contributed a recorded value to the cumulative-worst analysis at each landmark. Because grade 1 toxicity could not be consistently distinguished from no toxicity in retrospective clinical documentation, grade 0–1 toxicity was not reported separately; the table therefore presents grade 2 and grade ≥3 toxicity categories only. Abbreviations: CTCAE, Common Terminology Criteria for Adverse Events version 5.0; GU, genitourinary; GI, gastrointestinal.

Supplementary Table S3. Monthly landmark prevalence of grade ≥2 and grade ≥3 genitourinary and gastrointestinal toxicity by treatment platform over 24 months.

Month	G2+ GU MRgSBRT	G2+ GU CTgSBRT	G2+ GI MRgSBRT	G2+ GI CTgSBRT	G3+ GU MRgSBRT	G3+ GU CTgSBRT	G3+ GI MRgSBRT	G3+ GI CTgSBRT
0	0.0%	0.9%	0.0%	0.0%	0.0%	0.0%	0.0%	0.0%
1	35.4%	33.9%	2.0%	6.1%	2.0%	0.0%	1.0%	0.0%
2	37.4%	38.3%	3.0%	7.0%	2.0%	0.0%	1.0%	0.0%
3	35.7%	35.4%	3.1%	7.1%	1.0%	0.0%	1.0%	0.0%
4	26.5%	30.6%	1.0%	6.3%	1.0%	0.9%	0.0%	0.0%
5	28.6%	30.6%	2.0%	6.3%	1.0%	0.9%	0.0%	0.0%
6	26.0%	27.0%	3.1%	5.4%	1.0%	0.9%	0.0%	0.0%
7	27.4%	27.8%	3.2%	5.6%	1.1%	0.9%	0.0%	0.0%
8	24.7%	27.4%	3.2%	5.7%	1.1%	0.9%	0.0%	0.0%
9	23.3%	26.2%	3.3%	4.9%	1.1%	1.0%	0.0%	0.0%
10	21.2%	24.5%	3.5%	3.9%	1.2%	1.0%	0.0%	0.0%
11	19.8%	25.5%	2.5%	4.1%	1.2%	1.0%	0.0%	0.0%
12	17.1%	22.3%	2.6%	4.3%	1.3%	1.1%	0.0%	0.0%
13	19.7%	23.1%	2.8%	4.4%	1.4%	1.1%	0.0%	0.0%
14	20.6%	23.3%	1.6%	4.4%	1.6%	1.1%	0.0%	0.0%
15	19.7%	18.8%	1.6%	2.5%	1.6%	1.3%	0.0%	0.0%
16	20.0%	16.9%	1.8%	2.6%	1.8%	1.3%	0.0%	0.0%
17	18.9%	17.6%	1.9%	2.7%	1.9%	1.4%	0.0%	0.0%
18	18.2%	17.6%	2.3%	2.9%	0.0%	1.5%	0.0%	0.0%
19	10.0%	17.2%	2.5%	3.1%	0.0%	1.6%	0.0%	0.0%
20	8.6%	18.2%	2.9%	1.8%	0.0%	1.8%	0.0%	0.0%
21	10.7%	20.0%	3.6%	2.0%	0.0%	2.0%	0.0%	0.0%
22	13.0%	20.8%	4.3%	2.1%	0.0%	2.1%	0.0%	0.0%
23	13.6%	21.4%	4.5%	2.4%	0.0%	2.4%	0.0%	0.0%
24	15.0%	20.0%	5.0%	2.9%	0.0%	2.9%	0.0%	0.0%

Values are percentages of evaluable patients at each monthly landmark and represent toxicity present at that landmark, not cumulative worst toxicity. At each landmark, a toxicity was considered present if its onset date was on or before the landmark and it had not resolved before the landmark. Patients who died or were lost to follow-up before a landmark were excluded from the denominator. At the prespecified 3-, 12-, and 24-month landmarks, the numbers of evaluable patients were 211, 170, and 55 overall, respectively; corresponding CTgSBRT/MRgSBRT denominators were 113/98, 94/76, and 35/20. Abbreviations: CTCAE, Common Terminology Criteria for Adverse Events version 5.0; GU, genitourinary; GI,

gastrointestinal; MRgSBRT, MR-guided stereotactic body radiation therapy; CTgSBRT, CT-guided stereotactic body radiation therapy; G2+, grade ≥2; G3+, grade ≥3.
